# Adaptation in Predictive Prosodic Processing in Bilinguals

**DOI:** 10.3389/fpsyg.2021.661236

**Published:** 2021-05-28

**Authors:** Anouschka Foltz

**Affiliations:** Institute of English Studies, University of Graz, Graz, Austria

**Keywords:** predictive processing, prosody, contrastive accent, Bayesian adaptation model, prediction error

## Abstract

Native language listeners engage in predictive processing in many processing situations and adapt their predictive processing to the statistics of the input. In contrast, second language listeners engage in predictive processing in fewer processing situations. The current study uses eye-tracking data from two experiments in bilinguals’ native language (L1) and second language (L2) to explore their predictive processing based on contrastive pitch accent cues, and their adaptation in the face of prediction errors. The results of the first experiment show inhibition effects for unpredicted referents in both the L1 and the L2 that can be modeled with a Bayesian adaptation model, suggesting that bilinguals adapt their prediction in the face of prediction errors in a way that is compatible with the model. In contrast, the results of the second experiment, after a training phase that increased the predictive validity of the cue, show inhibition effects for unpredicted referents only in the L1, but not in the L2. In addition, the Bayesian adaptation model significantly predicts only the L1, but not the L2 data. The results are discussed with respect to adaptation to the statistical properties of the input.

## Introduction

Increasing evidence suggests that prediction (or anticipation) is an integral process during language comprehension in one’s native language (L1; Kamide, 2008; Altmann and Mirković, 2009). Native listeners use their prior linguistic and non-linguistic knowledge and experience to generate predictions about the unfolding input. As such, comprehenders are not merely passive adaptors to the input, but active contributors to the construction of meaning. For example, native listeners can employ different linguistic cues and real-world plausibility information to make predictions, such as verb meaning (Altmann and Kamide, 1999), linguistic gender (Dahan et al., 2000; Lew-Williams and Fernald, 2010), thematic dependencies (Kamide et al., 2003a), case marking (Kamide et al., 2003b), or prosody (Weber et al., 2006b). These kinds of cues allow listeners to predict information at different levels of linguistic representation, for example, lexical (DeLong et al., 2005; Van Berkum et al., 2005), syntactic category (Lau et al., 2006; Dikker et al., 2010), or case marking information (Weber et al., 2006b).

Prediction also plays a major role in current theories of language comprehension (Kuperberg and Jaeger, 2016). A particular focus in some theoretical approaches has been on prediction error or prediction failure. Specifically, prediction error is seen as one of the major mechanisms that allows us to update and adapt our predictions, thus leading to learning (Schultz and Dickinson, 2000; Clark, 2013; Dell and Chang, 2014). The extent of learning or adaptation is relative to the size of the prediction error, such that larger prediction errors lead to more learning or larger adaptation (Dell and Chang, 2014): The more unpredicted the input, the greater the update or learning. The size of the prediction error thus depends on how strong the initial prediction was, which in turn depends on both prior and recent experience with the input (Jaeger and Snider, 2013).

While there is ample evidence for predictive processing and adaptation in the face of prediction errors in the L1 (Kamide, 2008; Altmann and Mirković, 2009; Jaeger and Snider, 2013; Kuperberg and Jaeger, 2016), the role of predictive processing in a second language (L2) is not well-understood. While a large body of evidence suggests that L2 speakers engage in predictive processing to a lesser extent compared to native speakers (Kaan, 2014; Grüter et al., 2017), we so far have relatively little knowledge of the kinds of processing situations in which L2 learners would or would not engage in predictive processing and why this would be the case. There is also evidence that L2 learners adapt to the input differently in their L1 compared to their L2 (Foltz, 2020). One possibility is that L2 learners have difficulties in generating a prediction error when faced with unpredicted input, such that they adjust their predictions less than native speakers or not at all in the face of a prediction error. Another possibility is that L2 learners do generate a prediction error, but do not adjust their future predictions accordingly.

The current study uses a Bayesian adaptation model (Delaney-Busch et al., 2019; Ness and Meltzer-Asscher, 2021a), which includes trial-by-trial adaptation to the input and incorporates prediction error, to explore if such a model is compatible with the processing of prosodic cues observed in both the L1 and the L2. Specifically, the study uses the eye-tracking data from German-English bilinguals from Foltz (2020) to explore the hypotheses that a strong prediction error leads to delayed processing of the unpredicted input and causes adaptation in the forms of reduced prediction in future trials, such that the number of disconfirmed predictions affects processing.

### Contrastive Pitch Accents as a Cue to Prediction in the L1 and the L2

The predictive prosodic cue of interest in the current study is the so-called contrastive pitch accent (L+H* using ToBI labeling; cf., Silverman et al., 1992). In both German and English, a contrastive pitch accent is characterized by a low leading tone, followed by a steep rise in pitch to a high tone that is anchored to the stressed syllable of the word that carries the pitch accent. As the name implies, a contrastive pitch accent evokes a contrast set (Ito and Speer, 2008; Braun et al., 2019), such that the pitch-accented word contrasts with some other entity in the discourse. Importantly, adult native speakers of both German and English can use contrastive pitch accents to make predictions about upcoming referents in the discourse. For example, native English speakers listening to instructions, such as *Hang the blue angel. […] Now, hang the GREEN…* (where ALL CAPS indicate a L+H* accent), predicted that the noun *angel* will be repeated (Ito and Speer, 2008). Such predictive processing was not found if listeners instead heard *Hang the blue angel. […] Now, hang the green…* (with no L+H* accent; Ito and Speer, 2008, Experiment 2). Similarly, native German speakers listening to instructions, such as *Klick die lila Schere an*. *Klick die ROTE…* (literally: *Click the purple scissors on. Click the RED…*), predicted that the noun *Schere* (*scissors*) will be repeated. Such predictive processing was attenuated if listeners instead heard *Klick die lila Schere an*. *Klick die rote…* (literally: *Click the purple scissors on. Click the red…*; Weber et al., 2006a; Foltz, 2020). In both cases, the predictive L+H* cue occurred on the word immediately preceding the predicted noun, suggesting that native listeners can use the contrastive pitch accent cue rapidly to predict upcoming referents in the discourse.

In contrast, the available evidence suggests that L2 listeners do not always use contrastive pitch accents to predict upcoming referents (Klassen, 2015; Namjoshi, 2015; Takeda, 2018; Perdomo and Kaan, 2019; Foltz, 2020; Nakamura et al., 2020). Most relevantly for the current study, Foltz (2020) tested German-English bilingual listeners’ processing of contrastive pitch accents in both of their languages. She found that participants used L+H* accents to predict upcoming referents in their L1, but not consistently in their L2, even though both languages mark contrastiveness through the same L+H* cue. Specifically, Foltz (2020) found that L2 listeners initially showed no evidence for predictive processing, but for facilitative processing, where the pattern expected for predictive processing occurred only after identifying segmental information from the final noun had come in. Specifically, when hearing *Click on the red banana. Click on the GREEN banana*, participants looked at the green banana earlier than when hearing *Click on the red banana. Click on the green banana*, but this difference emerged only during the processing of the second instruction’s final noun *banana* and was, therefore, not predictive, but only facilitative, in nature. A second experiment found evidence for predictive processing in the same L2 listeners after a training phase in which listeners heard only felicitous L+H* accents (i.e., where a L+H* accent consistently preceded a repeated noun, increasing the predictive validity of the cue). Foltz (2020) also compared predictive processing in the first and second halves of both experiments. A decrease in predictive processing over the course of each experiment was expected because in half of the experimental trials with a L+H* accent on the adjective (e.g., *Click on the red banana. Click on the GREEN…*), participants heard the expected repeated noun (*banana*), but in the other half of the trials, they heard an unexpected novel noun (e.g., *duck*). During the experiment, the L+H* cue was thus inconsistently used and not very informative, such that listeners should decrease their predictive processing as evidence accumulates that predicting a repeated noun is as often wrong as it is right (Kuperberg and Jaeger, 2016). This indeed happened in the bilinguals’ L1, but not in their L2. While predictive processing decreased over the course of Experiment 1 in the L1, it increased in the L2. Following the training phase of Experiment 2 (which increased the predictive validity of the L+H* cue), predictive processing remained stable over the course of the experiment in the L1, but again increased in the L2. Overall, the bilinguals in the study by Foltz (2020) showed expected patterns of predictive processing in the L1, but not the L2.

### Prediction, Prediction Error, and Adaptation

As mentioned above, evidence for prediction in L1 language processing is pervasive (Kamide, 2008; Altmann and Mirković, 2009; Kuperberg and Jaeger, 2016). There is also plenty of evidence that prediction is graded rather than an all-or-nothing process, and that it is probabilistic (see Kuperberg and Jaeger, 2016, for an overview). This fits within a rational approach to cognition (Anderson, 1990), where comprehenders use all of their stored probabilistic knowledge as well as the preceding context to maximize the probability of accurate recognition during processing. Prediction also has implications for the allocation of resources during language processing. Without prediction, resources would need to be allocated directly in response to the properties of the input, and resource bottlenecks in comprehension may, for example, occur at word onsets as this is where most of the bottom-up “work” of lexical comprehension occurs (Delaney-Busch et al., 2019). Prediction allows us to distribute resources more evenly, for example, by predicting properties of an upcoming word, and thus decreasing potential resource bottlenecks at word onsets by using resources ahead of time to minimize resource use later. Specifically, by predicting a repeated noun, listeners can alleviate a potential resource bottleneck at the onset of this particular noun.

There is also increasing evidence for predictive pre-activation during language processing (see Kuperberg and Jaeger, 2016, for an overview). Predictive pre-activation occurs when comprehenders use high-level event hypotheses to activate linguistic representations before bottom-up input reaches these levels of representation. Predictive pre-activation is graded and the degree of pre-activation relates to the strength and specificity of the prediction (Ness and Meltzer-Asscher, 2018). Most relevantly for the current work, there is evidence that comprehenders use prosodic cues, such as L+H* accents, to form these high-level event hypotheses and predictively pre-activate particular lexical items before bottom-up input for these lexical items has come in (Weber et al., 2006a; Ito and Speer, 2008; Foltz, 2020). As mentioned above, these studies found that participants had increased looks to the picture of a green banana when hearing instructions like *Click on the red banana. Click on the GREEN…* compared to hearing instructions like *Click on the red banana. Click on the green…*. Importantly, these increased looks to the green banana occurred before bottom-up information from the following noun (*banana* or *duck*) had come in, suggesting that listeners had predictively pre-activated linguistic representations of this referent.

Predictive pre-activation can also lead to predictive pre-updating (Lau et al., 2013; Ness and Meltzer-Asscher, 2018), where listeners update and include the predicted content in the linguistic representation that they are building in working memory before receiving bottom-up input. While predictive pre-activation is graded, predictive pre-updating involves commitment and is thus an “all or nothing” process that occurs when the predicted content passes a particular threshold (Ness and Meltzer-Asscher, 2018, 2021b). Comprehenders are more likely to reach the threshold to predictively pre-update if they generate a stronger prediction, for example, when predictive validity is high or a context is highly constraining (Ness and Meltzer-Asscher, 2018). However, prediction also bears the risk of predicting incorrectly, thus generating a prediction error. In fact, this is costly in terms of processing resources, especially if listeners have formed a strong prediction and have pre-updated linguistic representations. Specifically, Ness and Meltzer-Asscher (2018, 2021b) assume that only pre-updating, but not pre-activation, would lead to additional processing costs following a disconfirmed prediction. In line with this, studies have found that disconfirmed strong predictions incur additional processing costs (Federmeier et al., 2007). Ness and Meltzer-Asscher (2018) have suggested that such processing costs stem from the inhibition of the predictively pre-updated representations, which is required to integrate the actual bottom-up input.

The probabilistic nature of prediction in language processing minimizes this risk because listeners can base their predictions on the statistical structures of the input as well as adapt their predictions when faced with changes in the statistical structures of the input (Delaney-Busch et al., 2019; Ness and Meltzer-Asscher, 2021a). In fact, there is evidence that listeners track the informativeness of linguistic cues, including prosodic cues, in the input and adapt their processing in response to the distributional properties and the reliability of these cues (Kurumada et al., 2014, 2018; Hopp, 2016; Roettger and Franke, 2019; Roettger and Rimland, 2020). For example, Kurumada et al. (2014) exposed listeners to either a reliable or unreliable speaker, i.e., a speaker whose use of L+H* accents either did or did not provide reliable information about upcoming referents. In a following test session, only participants who had heard the reliable speaker used L+H* information for reference identification, but participants who had heard the unreliable speaker did not, suggesting that native listeners considered the prior reliability of L+H* accents to inform their predictions during language processing. Similarly, participants in Roettger and Franke (2019) heard either a reliable speaker, whose prosodic patterns always matched the discourse context, or an unreliable speaker, whose prosodic patterns mismatched the discourse context in one third of the trials. Their results showed adaptation to consistent and inconsistent prosodic cues. Specifically, consistent use of initially weak prosodic cues can strengthen these cues over time, leading to earlier/stronger prediction, and inconsistent use of initially strong prosodic cues can weaken these cues over time, thus delaying/weakening prediction. Furthermore, Roettger and Rimland (2020) showed that listeners can adapt their use of prosodic cues in a speaker-specific manner, such that they can learn that a particular speaker consistently uses unconventional prosodic cues and then use those unconventional cues to predict upcoming referents when listening to that specific speaker.

### The Current Study

The current study explores how bilinguals adapt their processing of prosodic cues in both their L1 and their L2 when faced with strong prediction errors. To do so, the study uses a Bayesian adaptation model, based on Delaney-Busch et al. (2019) and Ness and Meltzer-Asscher (2021a), to explore if the model captures how participants update their beliefs about the predictive validity of prosodic cues to inform the strength of their future predictions. A Bayesian adaptation model was chosen because such models have been widely used to account for various linguistic phenomena (Lassiter and Goodman, 2015; Myslín and Levy, 2016; Delaney-Busch et al., 2019; Werning et al., 2019; Ness and Meltzer-Asscher, 2021a), including rational adaptation to prosodic cues (Roettger and Franke, 2019; Roettger and Rimland, 2020). Furthermore, the particular model chosen here explicitly models prediction error, which is assumed to drive adaptation and learning in many theoretical frameworks of language processing (Chang et al., 2006; Dell and Chang, 2014).

The current study expands previous results on trial-by-trial adaptation to the input in two ways. First, the current study uses a Bayesian adaptation model to model adaptation to prosodic cues in eye movement data. Previous studies have used similar models to explore adaptation in behavioral (response times), EEG, and mouse tracking data (Delaney-Busch et al., 2019; Roettger and Franke, 2019; Roettger and Rimland, 2020; Ness and Meltzer-Asscher, 2021a). Specifically, the model used in the current study explores potential trial-by-trial adaptation in response to the validity of contrastive pitch accents as a cue to predicting upcoming referents. Contrastive pitch accents are a good testing ground for potential adaptation to the input as a result of prediction error because listeners seem to generate strong predictions when encountering these accents.

Evidence for the predictive strength of L+H* accents comes from the speed with which this particular predictive cue is used in processing as well as the large prosodic garden-path effect that is observed when predictions based on the processing of L+H* accents are not confirmed (Weber et al., 2006a; Ito and Speer, 2008). Furthermore, production data for English (Ito and Speer, 2006) suggest that L+H* accents in a discourse context similar to *Click on the red banana. Click on the GREEN…* consistently co-occur with a following repeated noun (*banana*), and thus consistently mark the color contrast. Specifically, participants in Ito and Speer (2006) instructed each other to hang different-colored objects on a Christmas tree, eliciting contrastive and non-contrastive adjective-noun sequences similar to the instructions used in Weber et al. (2006a), Ito and Speer (2008), and Foltz (2020). English adjectives in a non-contrastive discourse context, such as an instruction to hang a white hat followed by an instruction to hang a blue house on the Christmas tree, carried a L+H* accent in only 3–4% of cases. In other words, non-contrastive adjectives are almost never marked with a L+H* accent, providing a reliable cue that the presence of a L+H* accent marks a contrast. Conversely, marking the color contrast with a L+H* accent is optional, such that English adjectives in a contrastive discourse context, such as an instruction to hang a white house followed by an instruction to hang a blue house on the Christmas tree, still carried a L+H* accent less than 50% of the time. German production data from a discourse context similar to *Click on the red banana. Click on the GREEN…* are not available, but German production data from a reading task, where participants were instructed to speak as naturally as possible, suggest a similar distribution of L+H* accents in German, with 8% of non-contrastive items receiving a L+H* accent and 50% of contrastive items carrying a L+H* accent (Sudhoff, 2010). In other words, in both languages the presence of a L+H*-accented adjective strongly suggests a contrast and a following repeated noun, but the absence of a L+H*-accented adjective is found frequently in both contrastive and non-contrastive situations. Prior experience should thus lead listeners to make strong predictions of a following repeated noun when encountering a L+H* accent in this particular context, but not when not encountering a L+H* accent. In other words, a novel noun following a L+H*-accented adjective should be highly unexpected and should generate a large prediction error.

Second, and more importantly, the current study expands the use of a Bayesian adaptation model that models prediction error to L2 predictive processing. L2 listeners engage in predictive processing in fewer processing situations than L1 listeners (Kaan, 2014; Grüter et al., 2017), and seem not to use the prosodic cues for prediction as effectively as native listeners (Klassen, 2015; Namjoshi, 2015; Takeda, 2018; Perdomo and Kaan, 2019; Foltz, 2020). The current study, therefore, explores if a Bayesian adaptation model can account for changes in eye movement behavior in L1 and L2 processing across two experiments which differ in the initial reliability of predictive cues. Specifically, the second experiment was preceded by a training phase, where L+H* accents acted as a consistent cue to upcoming referents, which was expected to facilitate predictive processing. Thus, the study explores whether the model fits the L1 and L2 data, which would provide further evidence for the role of prediction error in adaptation and learning during L1 and L2 processing.

The study uses the eye-tracking data from German-English bilinguals from the study by Foltz (2020). Foltz (2020) conducted a Smoothing Spline ANOVA analysis (Gu, 2013) to establish if evidence for predictive processing occurred during the processing of the color adjective (*GREEN*) in sequences like *Click on the red banana. Click on the GREEN…*, i.e., before disambiguating information from the following noun had come in. The focus in Foltz (2020) as well as other previous studies (e.g., Weber et al., 2006a; Ito and Speer, 2008) was thus on processing patterns while processing the adjective. In contrast, the current study focuses on prediction error and the adaptation in processing that a large prediction error may cause, and, therefore, on processing patterns while processing the disambiguating noun. Specifically, while the prediction itself should be generated upon encountering the L+H* cue on the adjective, a prediction error should be generated upon encountering a noun that does not match the prediction. Overall, the current study explores the hypotheses that a strong prediction error leads to delayed processing of the unpredicted input and causes adaptation in the form of reduced prediction in future trials, such that the number of disconfirmed predictions affects processing.

## Methods

Data from the two experiments reported in Foltz (2020) are analyzed for the current paper. The methods are described in detail in Foltz (2020) and will be summarized here.

### Participants

Participants were 17 native-German intermediate-to-advanced (B2 or above using CEFR levels; Council of Europe, 2001) learners of English (four male, 13 female; mean age 24.5, *SD* = 5.2), who had been learning English for an average of 10.9 years (*SD* = 2.9). One additional participant was excluded for having too many missing data points due to track loss (37% vs. under 20% for all other participants). Participants self-rated their English proficiency on a scale from 1 being beginner to 5 being native, with the following average ratings: reading (mean = 3.3, *SD* = 0.8), writing (mean = 2.7, *SD* = 0.7), comprehension (mean = 3.0, *SD* = 0.7), and speaking (mean = 2.6, *SD* = 0.9).

### Materials and Procedure

**Figure 1:**
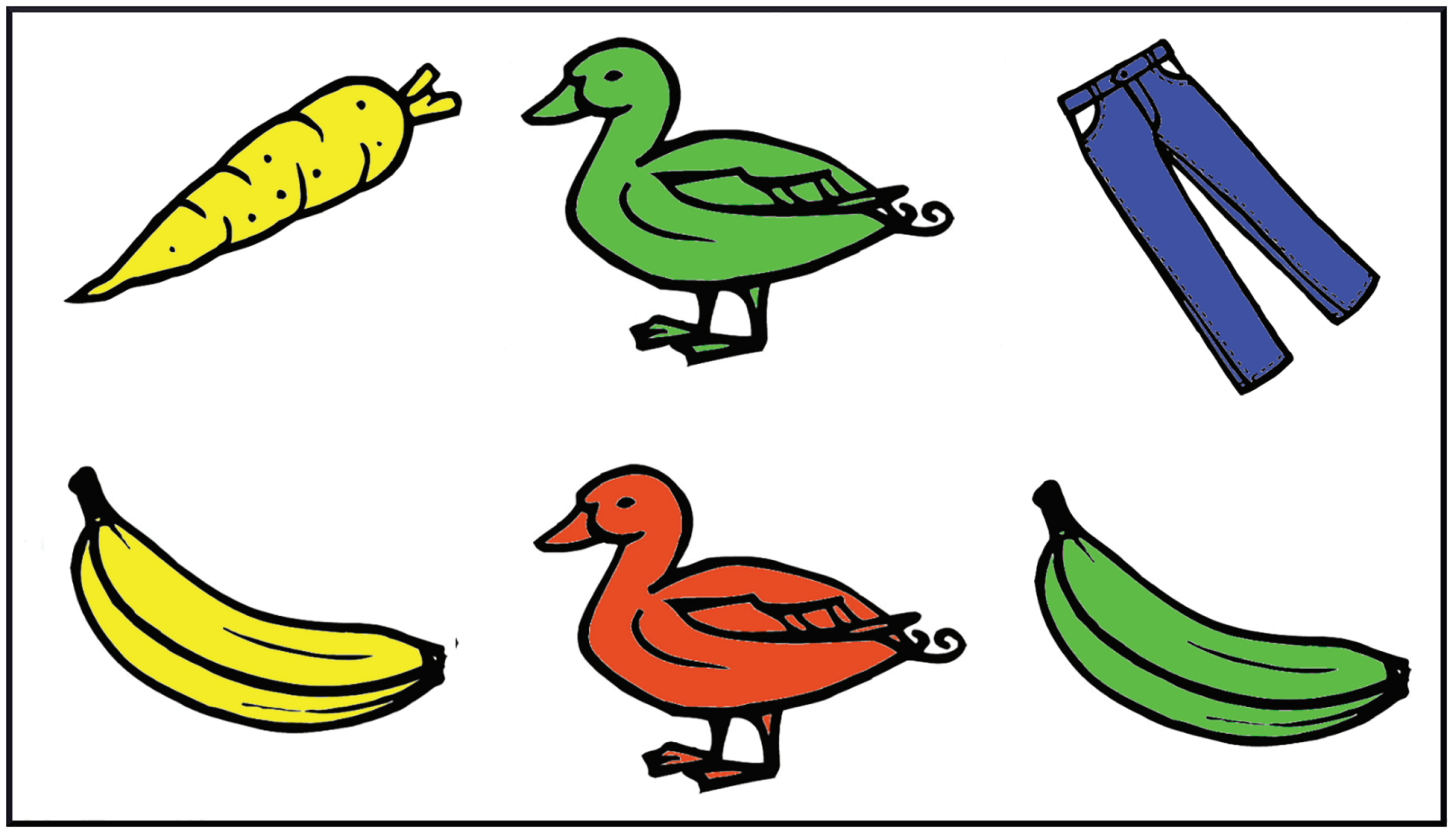
Sample experimental display. Objects pictured were adapted from materials by Saskia, Gast, and Janina Valko and are available at madoo.net under a (cc) Creative Commons by-sa license.

Each participant took part in two 48-trial experiments in their L1 German and in identical experiments in their L2 English. Both experiments used the same materials, which consisted of colored line drawings of various objects and pre-recorded instructions to click on these objects. There were 24 different objects, each colored in four different colors (blue, green, red, and yellow) and assigned to one of six picture sets, such that each set contained four different objects in four different colors. In any given trial, participants saw six colored objects (in two rows with three objects each) from the same picture set on a computer screen (see Figure 1). The German names for all objects in a set had the same grammatical gender, so that for each trial, listeners could not use gender information to predict upcoming referents (Lew-Williams and Fernald, 2010). The location on the screen of individual objects was varied for each trial, so that no visual displays were identical across the experiment.

A balanced German-English bilingual produced instructions of the form *Click on the [color] [object name]*, such as *Click on the green banana* or *Klick die grüne Banane an* (literally: *Click the green banana on*), for all objects in all four colors in both German and English. She further produced each instruction with three prosodic patterns: a clear L+H* accent on the color adjective, a clear L+H* accent on the noun, or no L+H* accent, which typically resulted in a H* accent on the color adjective and a !H* accent on the noun (see Foltz, 2020, for acoustic analyses confirming the prosodic patterns), for a total of 576 utterances (24 objects x 4 colors x 3 prosodic patterns x 2 languages).

During each trial, participants heard two instructions to click on two of the six objects on the screen. Instruction pairs had the following lexical contrasts: a repeated noun (e.g., red banana – green banana), a repeated adjective (e.g., green duck – green banana), or no repeated lexical item (e.g., red duck – green banana). The first instruction in each pair was always produced with no L+H* accent, and the second instruction was produced with one of three different prosodic patterns: a L+H* accent on the adjective (e.g., *GREEN banana*), a L+H* accent on the noun (e.g., *green BANANA*), or no L+H* accent (e.g., *green banana*). These lexical and prosodic properties of the instruction pairs were combined to create the different experimental and filler conditions. Table 1 shows the conditions that are relevant for the analyses in the current study. Specifically, the current analyses consider all trials (regardless of whether they were target or filler trials in Foltz, 2020) in which the two instructions involve two different color adjectives, i.e., all trials with a repeated noun or no repeated lexical item. Only trials involving two different color adjectives are considered here because a non-repeated color adjective is a prerequisite for predicting that the two successive instructions to click on objects differ only in the color adjective, and thus contain a repeated noun. Instruction pairs with a repeated color adjective were thus excluded from the current analyses. For all conditions involved in the current analyses, the display shown on the computer screen always included the object mentioned in the first instruction (e.g., a red duck), the same object in a different color (e.g., a green duck), and another object in that color (e.g., a green banana), one of which would be mentioned in the second instruction.

**Table 1:** Relevant conditions for the current analyses. The second sentence in each instruction pair is the target sentence.

Predictive cue	Repeated noun	Example instruction pair	Number of trials
Yes	Yes	Click on the red banana. Click on the GREEN banana.	6
	No	Click on the red duck. Click on the GREEN banana.	6
No	Yes	Click on the red banana. Click on the green banana.	6
	No	Click on the red duck. Click on the green banana/BANANA.	18 (12/6)

The German and English versions of the experiments had the same order of trials to compare participants’ processing when engaged in exactly the same task that differed only in the language of the instructions. Participants took part in the two consecutive experiments in German and in English on different days, about a week apart, with language counterbalanced across participants (such that half of the participants took part in the two German experiments on the 1st day and the two English experiments on the 2nd day and vice versa for the other half of participants). Their eye movements were recorded with a Tobii Pro X2-60 remote eye tracker, and visual displays were shown on a Dell 25-inch monitor. For each experiment, participants were told that they would listen to instructions to click on objects on the computer screen, and that they should simply follow the instructions and click on the mentioned objects. In each trial, a blank screen lasted for 250 ms before the array of six objects appeared. After 200 ms of preview time, participants heard the first instruction. The second instruction began 200 ms after participants had clicked on the first object. Once participants had clicked on the second object, the next trial began.

Each experiment had a total of 48 trials (the 36 trials listed in Table 1 as well as 12 trials with a repeated adjective, which are excluded from the current analyses) with lexical contrasts and picture sets distributed across each experiment in a Latin square design and prosodic patterns distributed pseudorandomly, so that no two consecutive trials had the same pattern. Each experiment included six trials with a predictive L+H* accent on the adjective followed by a repeated noun (e.g., *Click on the red banana. Click on the GREEN banana*) and six trials with a predictive L+H* accent on the adjective followed by a novel noun (e.g., *Click on the red duck. Click on the GREEN banana*). In both cases, participants should strongly predict a repeated noun, such that a confirmed prediction involves a small prediction error and a disconfirmed prediction leads to a large prediction error. The remaining 24 trials that are relevant for the current analyses contained no L+H* accent on the adjective, and either a repeated or a novel noun. Since these trials do not involve any predictive prosodic cues (i.e., since these trials do not contain any L+H* accent on the adjective), no prosody-based prediction of a repeated noun is expected. The second experiment in each language immediately followed the first experiment and was additionally preceded by a 24-trial training phase in which participants heard 18 felicitous trials (12 *red duck – green banana* and six *green duck – green BANANA*) with no L+H* on the adjective (where no predictive prosodic processing is expected) and six felicitous trials (*red banana – GREEN banana*) with a L+H* on the adjective followed by a repeated noun (where in the vast majority of trials, participants should correctly predict a repeated noun, which yields a small prediction error to account for the few cases where participants may incorrectly predict a novel noun). The training phase contained the same proportion of trials with a L+H* on the adjective (0.25) as the following experimental phase to keep the phases as similar as possible and to avoid potential strategic behavior as a result of participants explicitly detecting differences across the two phases. The purpose of the training phase was to increase the predictive validity of the L+H* cue by presenting no trials that were expected to generate a strong prediction error.

### Analysis

For each of the two experiments (no training phase vs. training phase) in each of the two languages (L1 German and L2 English), the following analyses will be conducted on an analysis window ranging from 150 to 450 ms after the beginning of the noun. As it takes around 150–200 ms to plan and execute an eye movement (Fischer, 1998), this window corresponds approximately to the first 300 ms of processing the target noun. A 300 ms window was chosen to ensure that it reflected unambiguous processing of the target noun. All analyses included each trial that involved two different color adjectives (see explanation in section “Materials and Procedure”) and trials with repeated color adjectives, which all contained felicitous prosodic patterns, were excluded from the analyses (see section “Materials and Procedure”). All fixed effects in the analyses were centered to minimize collinearity and sum-coded for ANOVA-style main effects and interactions.

First, mixed logit models (A models; Baayen, 2008) investigate whether the experimental conditions of Predictive Cue (yes vs. no) and Repeated Noun (yes vs. no) as well as Trial Number (numeric) and all their interactions are significant predictors of listeners’ looks to the target object while processing the target noun. Trial number was included in this analysis and the third analysis (described below) because participants may get faster at the task over the course of the experiment or alternatively get slower over the course of the experiment due to fatigue and any effects of the Bayesian adaptation model should occur beyond such potential task effects. Initial models contain the maximal random effects structure (Barr et al., 2013), which is simplified until the model converges. Most importantly, the following effect is expected: if a L+H* accent leads to a strong prediction of a repeated noun, then the presence of this predictive cue followed by an expected repeated noun should lead to facilitation and increased looks to the target noun relative to encountering a repeated noun without the prior predictive cue. If strong predictions lead to strong inhibition of the alternative, then the presence of a predictive cue followed by an unexpected novel noun should lead to decreased looks to the target noun relative to a novel noun without the prior predictive cue. Thus, the analyses should yield a significant Predictive Cue by Repeated Noun interaction, which will then be further explored.

Second, a Bayesian adaptation model that models inhibition cost due to predictive pre-updating on a trial-by-trial basis (Delaney-Busch et al., 2019; Ness and Meltzer-Asscher, 2021a) was formulated to see if it is a significant predictor of looks to the target object by itself (B models). Following Ness and Meltzer-Asscher (2021a), inhibition cost for each trial was calculated as μ*PE, where μ represents listeners’ current belief of predictive validity and PE presents the prediction error.

More specifically, μ is a point estimate of predictive validity, defined as the mean of a beta distribution of participants’ beliefs about the likelihood of encountering the predicted referent: μ = mean of β(1 + number of L+H* on the adjective with repeated noun trials encountered, 1 + number of L+H* on the adjective with novel noun trials encountered). The initial prior in Experiment 1 is β(1, 1), and updating occurs whenever participants encounter a predictive L+H* accent on the adjective of the second color adjective (i.e., on *GREEN* in *Click on the red banana. Click on the GREEN…*). Since Experiment 2 in each language occurred immediately after Experiment 1 and the training session, the calculations of μ for Experiment 2 include the number of trials with a L+H* on the adjective and a repeated noun and the number of trials with a L+H* and a novel noun that participants encountered in Experiment 1 and in the training session.

PE is the prediction error. For trials in which participants encountered a L+H* accent, participants were expected to strongly predict a repeated noun (e.g., red banana – GREEN banana). Production data for English suggest that in only 4% of cases in a similar discourse context is a L+H*-accented adjective not contrastive (Ito and Speer, 2006). PE for the English experiments was, therefore, set to 0.04 for trials in which a L+H* accent on the adjective was followed by a repeated noun, and to 0.96 for trials in which a L+H* accent on the adjective was followed by a novel noun, thus generating a large prediction error in the latter case. Production data for German, albeit in a different discourse context, suggest that in only 8% of cases is a L+H*-accented item not contrastive (Sudhoff, 2010). PE for the German experiments was, therefore, set to 0.08 for trials in which a L+H* accent on the adjective was followed by a repeated noun, and to 0.92 for trials in which a L+H* accent on the adjective was followed by a novel noun, again generating a large prediction error in the latter case. PE was set to 0 for trials without a L+H* accent because there is no predictive prosodic cue and thus no prosody-based prediction error is expected. This aligns with previous production data, where a majority of contrastive and non-contrastive utterances in German and English carry no L+H* accent on the adjective (Ito and Speer, 2006; Sudhoff, 2010). Furthermore, while Roettger and Franke (2019) showed that weak prosodic cues (which the absence of a L+H* accent in the current study would arguably constitute) can eventually lead to prediction if used consistently, this is not the case here. Instead, adjectives both with and without a L+H* accent are followed equally often by a repeated noun as by a novel noun. It is, therefore, assumed here that listeners do not generate a prediction, and consequently no prediction error, for target nouns during trials with no L+H* predictive cue.

Inhibition Cost (μ*PE) was calculated for all included trials. In the formula, μ captures participants’ beliefs about the likelihood of encountering the predicted referent. Whenever participants encounter a L+H*-accented adjective with a repeated noun, the estimated predictive validity increases, and whenever participants encounter a L+H*-accented adjective with a novel noun, the estimated predictive validity decreases. Inhibition Cost is higher, the more L+H*-accented adjectives with a repeated noun participants have encountered and the more unexpected the noun is. The more L+H*-accented adjectives with a repeated noun participants have previously encountered, the more they can assume that a L+H* accent reliably cues a repeated noun in the current discourse situation, increasing prediction strength. This increases the Inhibition Cost because the stronger the initial prediction, the harder it is to then inhibit the predicted noun and integrate an unpredicted noun instead. Similarly, the more L+H*-accented adjectives with a novel noun participants have previously encountered, the less they can assume that a L+H* accent reliably cues a repeated noun, decreasing prediction strength. This decreases the Inhibition Cost because the weaker the initial prediction, the easier it is to then inhibit the predicted noun and integrate an unpredicted noun instead.

PE in the formula is the prediction error, which here is based on production data, i.e., on expected long-term frequencies in the input. The more likely it is to encounter a L+H*-accented adjective followed by a repeated noun in the language, the stronger the prediction to encounter a L+H*-accented adjective followed by a repeated noun and the larger the prediction error when this does not occur. A larger prediction error increases the Inhibition Cost because it is harder to inhibit a predicted noun and integrate an unpredicted noun instead if the prediction to encounter a L+H*-accented adjective followed by a repeated noun was strong. Thus, the formula to calculate Inhibition Cost takes into account both the dynamics in the current discourse situation (through μ) and the long-term distributional properties of the cue in the input (through PE).

**Figure 2:**
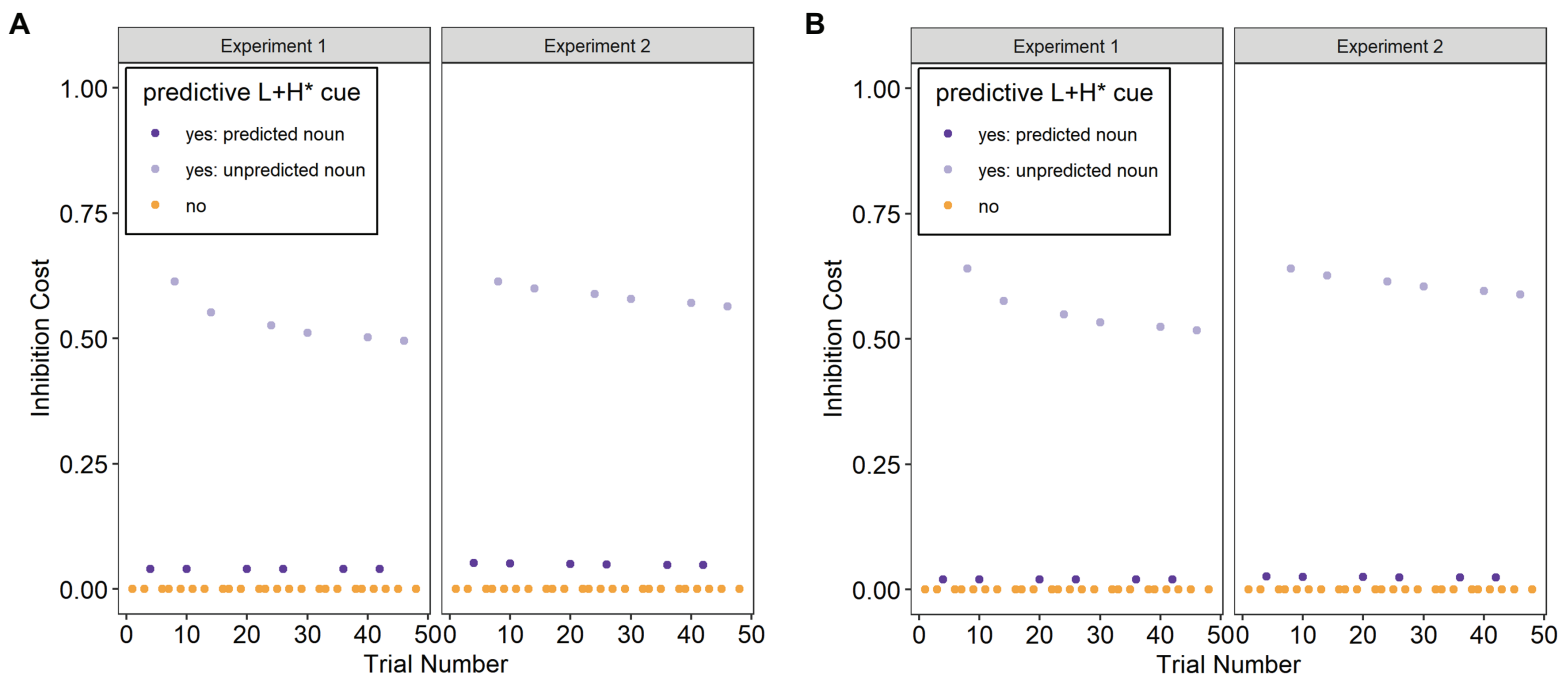
Modeled inhibition cost at each trial for Experiments 1 and 2 for **(A)** the L1 German and **(B)** the L2 English.

Figure 2 shows the calculated Inhibition Cost for both Experiment 1 (without a training session that increased the predictive validity of the L+H* cue) and Experiment 2 (with a training session that increased the predictive validity of the L+H* cue). The figure shows that the Inhibition Cost for unpredicted trials is initially similar across Experiments 1 and 2, but then drops off more gradually for Experiment 2 compared to Experiment 1. The Inhibition Cost for unpredicted trials decreases over the course of the experiments because participants adapt to the unreliability of the L+H* cue in experimental trials. This occurs more slowly in Experiment 2 because the training session increased the predictive validity of the prosodic cue, so that adaptation in the face of prediction errors is slower.

Third, if Inhibition Cost as modeled by the Bayesian adaptation model was a significant contributor by itself in the second analysis (B models), it was additionally included as a predictor in the original mixed logit model (C models) to see if it also significantly predicts looks to the target object and accounts for the trial-by-trial data beyond the experimental conditions Predictive Cue and Repeated Noun as well as Trial Number. The data and analysis scripts can be found on the Open Science Framework at https://osf.io/xey27/.

## Results

### Experiment 1: No Training

The current section presents the results from the German and English experiments that were not preceded by a training session.

#### L1 German

**Figure 3:**
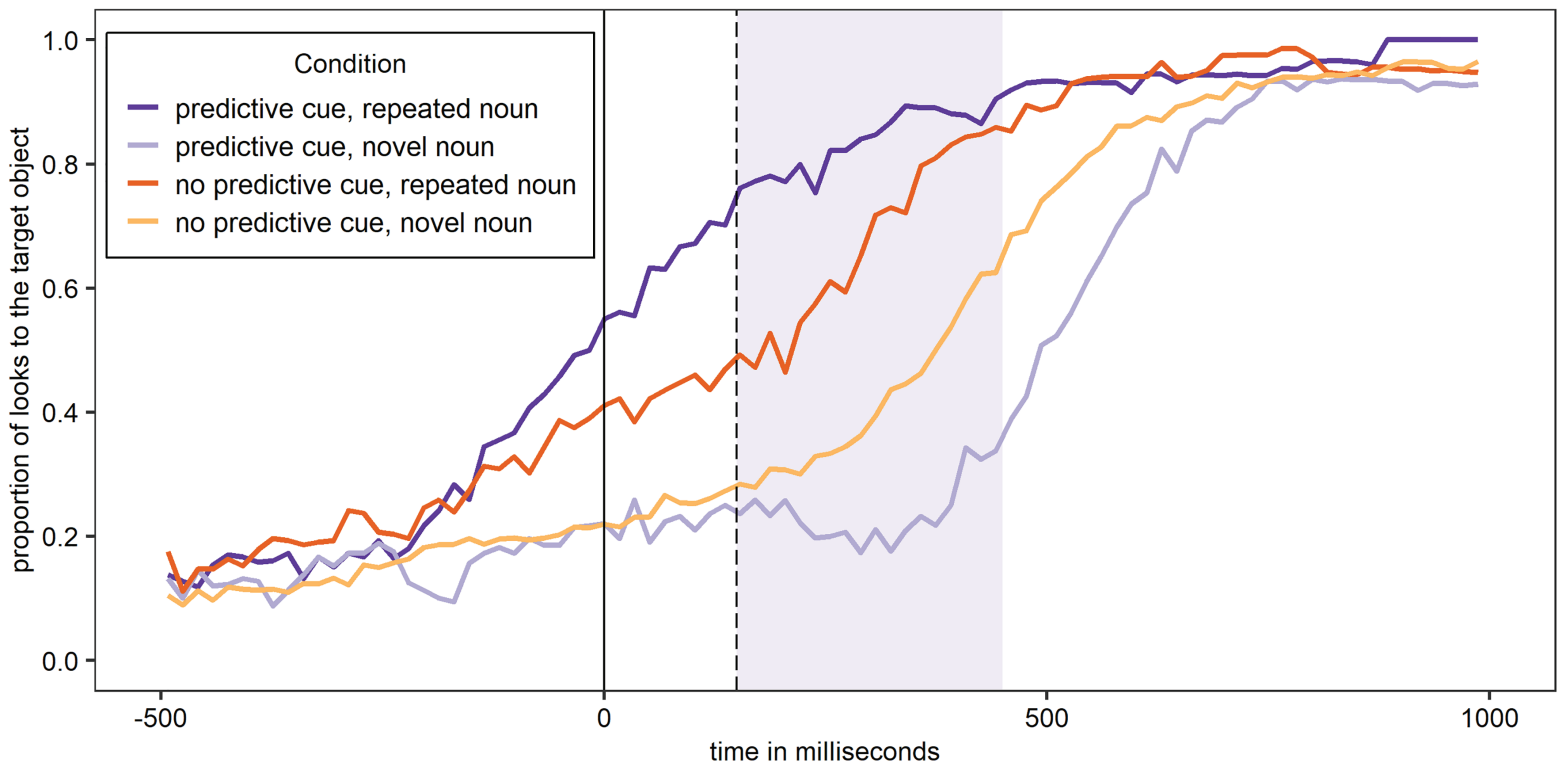
Proportion of looks over time to the target object across conditions in German (L1). The shaded area corresponds to the analysis window.

Figure 3 shows the proportion of looks to the target object over time (calculated as looks to the target object as numerator and looks to all six objects shown on the screen as denominator) for Experiment 1 and participants’ L1 German. In all figures, the relevant window for the data analysis is shaded. As expected, the figure shows most looks to the target object when a predictive L+H* cue was present and the noun was – as predicted – repeated, and the least looks to the target object when a predictive L+H* cue was present and the noun was – unexpectedly – not repeated.

The first analysis (Model 1A) tested if the experimental conditions and trial number significantly affected looks to the target object during the first 300 ms of processing the target noun in participants’ L1 German. The random effects structure of the final model included random intercepts for Participant and by-Participant random slopes for Predictive Cue, Repeated Noun, and Trial Number. The results are shown in Table 2 and reveal a significant main effect of Repeated Noun, such that there were significantly more looks to the target noun overall if the noun was repeated. Importantly, the Predictive Cue by Repeated Noun interaction was significant. To explore this interaction, separate models were fit for trials with repeated nouns and novel nouns, both with Predictive Cue as fixed factor, random intercepts for Participant, and by-Participant random slopes for Predictive Cue. The main effect of Predictive Cue did not quite reach significance for repeated nouns (β = −0.33, *SE* = 0.20, z = −1.65, *p* = 0.099), suggesting that the facilitation for a repeated noun from a L+H* predictive cue is merely numeric. In contrast, there was a significant main effect of Predictive Cue for novel nouns (β = 0.81, *SE* = 0.27, z = 3.05, *p* = 0.002^**^), providing evidence for inhibition of a novel noun following a predictive L+H* cue. Predictive Cue also significantly interacted with Trial Number, but this interaction will not be further explored here.

**Table 2:** Results of Models 1A, 1B, and 1C.

Fixed factors	Estimate	*SE*	*t-value*	*Value of p*
Model 1A				
Predictive Cue	0.29	0.18	1.58	=0.114
Repeated Noun	−1.12	0.25	−4.51	<0.001^***^
Trial Number	−0.25	0.14	−1.77	=0.077
Predictive Cue × Repeated Noun	0.45	0.04	12.78	<0.001^***^
Predictive Cue × Trial Number	0.16	0.04	4.60	<0.001^***^
Repeated Noun × Trial Number	0.03	0.03	0.96	=0.337
Predictive Cue × Repeated Noun × Trial Number	0.05	0.04	1.36	=0.175
Model 1B				
Inhibition Cost	−0.73	0.19	−3.78	<0.001^***^
Model 1C				
Predictive Cue	−2.48	0.73	−3.41	<0.001^***^
Repeated Noun	0.43	0.36	1.20	=0.230
Trial Number	−0.31	0.05	−6.57	<0.001^***^
Inhibition Cost	−3.00	0.85	−3.54	<0.001^***^
Predictive Cue × Repeated Noun	−1.35	0.49	−2.74	=0.006^**^
Predictive Cue × Trial Number	0.30	0.06	4.99	<0.001^***^
Repeated Noun × Trial Number	−0.08	0.04	−1.84	=0.065
Predictive Cue × Repeated Noun × Trial Number	0.20	0.05	4.07	<0.001^***^

The second analysis (Model 1B) tested whether the Bayesian adaptation model significantly predicts looks to the target object during the first 300 ms of processing the target noun. The random effects structure of the final model included random intercepts for Participant and by-Participant random slopes for Inhibition Cost. Table 2 shows that Inhibition Cost is indeed a significant predictor of participants’ looks to the target object. The larger the inhibition cost (as a result of a strong disconfirmed prediction), the fewer looks to the target object occurred, suggesting that participants did indeed inhibit the target noun if it was highly unexpected, and adapted their processing as modeled by the Bayesian adaptation model.

Finally, the third analysis (Model 1C) tested whether the Bayesian adaptation model can predict looks to the target object beyond the experimental conditions and the trial number. The random effects structure of the final model included only random intercepts for Participant. Table 2 shows that Inhibition Cost does indeed predict participants’ looks to the target object beyond the experimental conditions and the trial number. Again, the larger the inhibition cost, the fewer looks to the target object occurred. In order to see if Model 1C provided a better fit of the data compared to the same model without Inhibition Cost as a fixed effect, these two models were compared using the anova() function. Model 1C did indeed provide a significantly better fit of the data than the same model without Inhibition Cost (*p* < 0.001^***^; BIC Model 1C: 8156 vs. BIC model without Inhibition Cost: 8161; AIC Model 1C: 8088 vs. AIC model without Inhibition Cost: 8099).

#### L2 English

**Figure 4:**
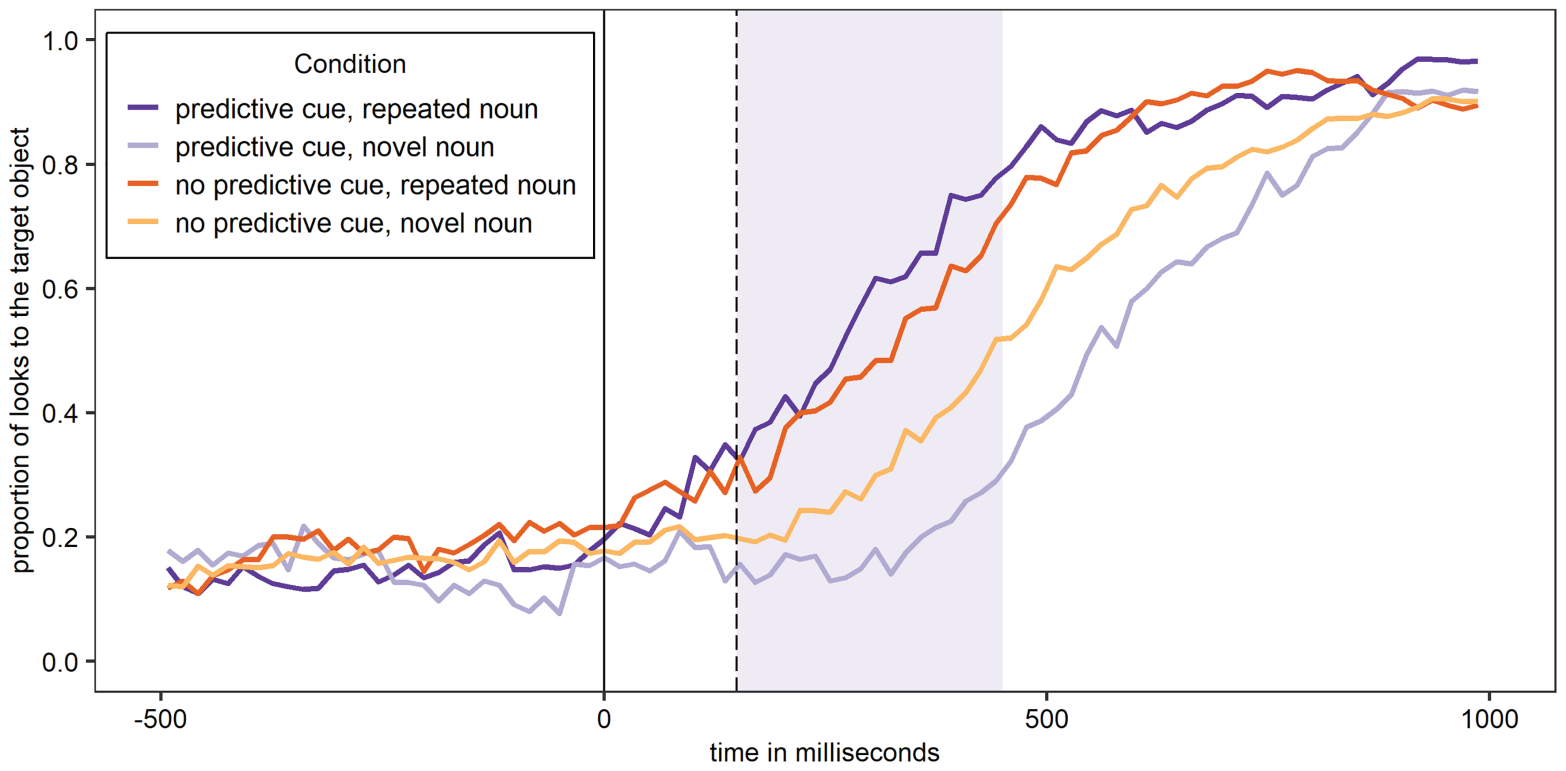
Proportion of looks over time to the target object across conditions in English (L2). The shaded area corresponds to the analysis window.

Figure 4 shows the proportion of looks to the target object over time for Experiment 1 and participants’ L2 English. As expected, the figure shows most looks to the target object when a predictive L+H* cue was present and the noun was – as predicted – repeated, and the least looks to the target object when a predictive L+H* cue was present and the noun was – unexpectedly – not repeated. While the overall pattern shown in Figure 4 for participants L2 English is the same as in Figure 3 for participants L1 German, the curves in Figure 4 cluster more closely together.

The first analysis (Model 2A) tested if the experimental conditions and trial number significantly affected looks to the target object during the first 300 ms of processing the target noun in participants’ L2 English. The random effects structure of the final model included random intercepts for Participant and by-Participant random slopes for Predictive Cue, Repeated Noun, and Trial Number. The results are shown in Table 3 and reveal a significant main effect of Predictive Cue, such that there were significantly more looks to the target object if there was no predictive L+H* cue compared to if there was. As in the analysis for the L1 German data, there was a significant main effect of Repeated Noun with significantly more looks to the target noun overall if the noun was repeated. Importantly, the Predictive Cue by Repeated Noun interaction was again significant. To explore this interaction, separate models were fit for trials with repeated nouns and novel nouns, both with Predictive Cue as fixed factor, random intercepts for Participant, and by-Participant random slopes for Predictive Cue. There was a main effect of Predictive Cue for both repeated nouns (β = −0.22, *SE* = 0.10, z = −2.34, *p* = 0.019^*^) and novel nouns (β = 0.62, *SE* = 0.23, z = 2.74, *p* = 0.006^**^). This provides evidence for both facilitation of a repeated noun and inhibition of a novel noun following a predictive L+H* cue. Predictive Cue also significantly interacted with Trial Number, and the three-way interaction was significant, but these interactions will not be further explored.

**Table 3:** Results of Models 2A, 2B, and 2C.

Fixed factors	Estimate	*SE*	*t-value*	*Value of p*
Model 2A				
Predictive Cue	0.17	0.08	2.02	=0.043^*^
Repeated Noun	−0.57	0.12	−4.82	<0.001^***^
Trial Number	−0.20	0.11	−1.86	=0.063
Predictive Cue × Repeated Noun	0.27	0.03	8.88	<0.001^***^
Predictive Cue × Trial Number	−0.09	0.03	−2.53	=0.011^*^
Repeated Noun × Trial Number	−0.02	0.03	−0.77	=0.440
Predictive Cue × Repeated Noun × Trial Number	0.27	0.03	8.73	<0.001^***^
Model 2B				
Inhibition Cost	−0.57	0.15	−3.86	<0.001^***^
Model 2C				
Predictive Cue	−5.58	0.85	−6.55	<0.001^***^
Repeated Noun	2.28	0.42	5.46	<0.001^***^
Trial Number	−0.41	0.05	−7.94	<0.001^***^
Inhibition Cost	−6.55	0.98	−6.70	<0.001^***^
Predictive Cue × Repeated Noun	−3.65	0.58	−6.31	<0.001^***^
Predictive Cue × Trial Number	0.36	0.07	5.34	<0.001^***^
Repeated Noun × Trial Number	−0.25	0.04	−5.93	<0.001^***^
Predictive Cue × Repeated Noun × Trial Number	0.53	0.05	10.30	<0.001^***^

The second analysis (Model 2B) tested whether the Bayesian adaptation model significantly predicts looks to the target object during the first 300 ms of participants processing the target noun in their L2 English. The random effects structure of the final model included random intercepts for Participant and by-Participant random slopes for Inhibition Cost. Table 3 shows that, as for participants’ L1 German, Inhibition Cost is indeed a significant predictor of participants’ looks to the target object in their L2 English. Again, participants inhibited the target noun if it was highly unexpected and adapted their processing as modeled in the Bayesian adaptation model.

Finally, the third analysis (Model 2C) tested whether the Bayesian adaptation model can predict looks to the target object beyond the experimental conditions and the trial number. The final model included no random effects. Table 3 shows that Inhibition Cost does indeed predict participants’ looks to the target object beyond the experimental conditions and the trial number. Again, the larger the inhibition cost, the fewer looks to the target object occurred. In order to see if Model 2C provided a better fit of the data compared to the same model without Inhibition Cost as a fixed effect, these two models were compared using the anova() function. Model 2C did indeed provide a significantly better fit of the data than the same model without Inhibition Cost (*p* < 0.001^***^; BIC Model 2C: 8483 vs. BIC model without Inhibition Cost: 8526; AIC Model 2C: 8421 vs. AIC model without Inhibition Cost: 8471).

### Experiment 2: After Training

The current section presents the results from the German and English experiments that were preceded by a training session that increased the predictive validity of the L+H* cue. In each language, these experiments occurred immediately after Experiment 1.

#### L1 German

**Figure 5:**
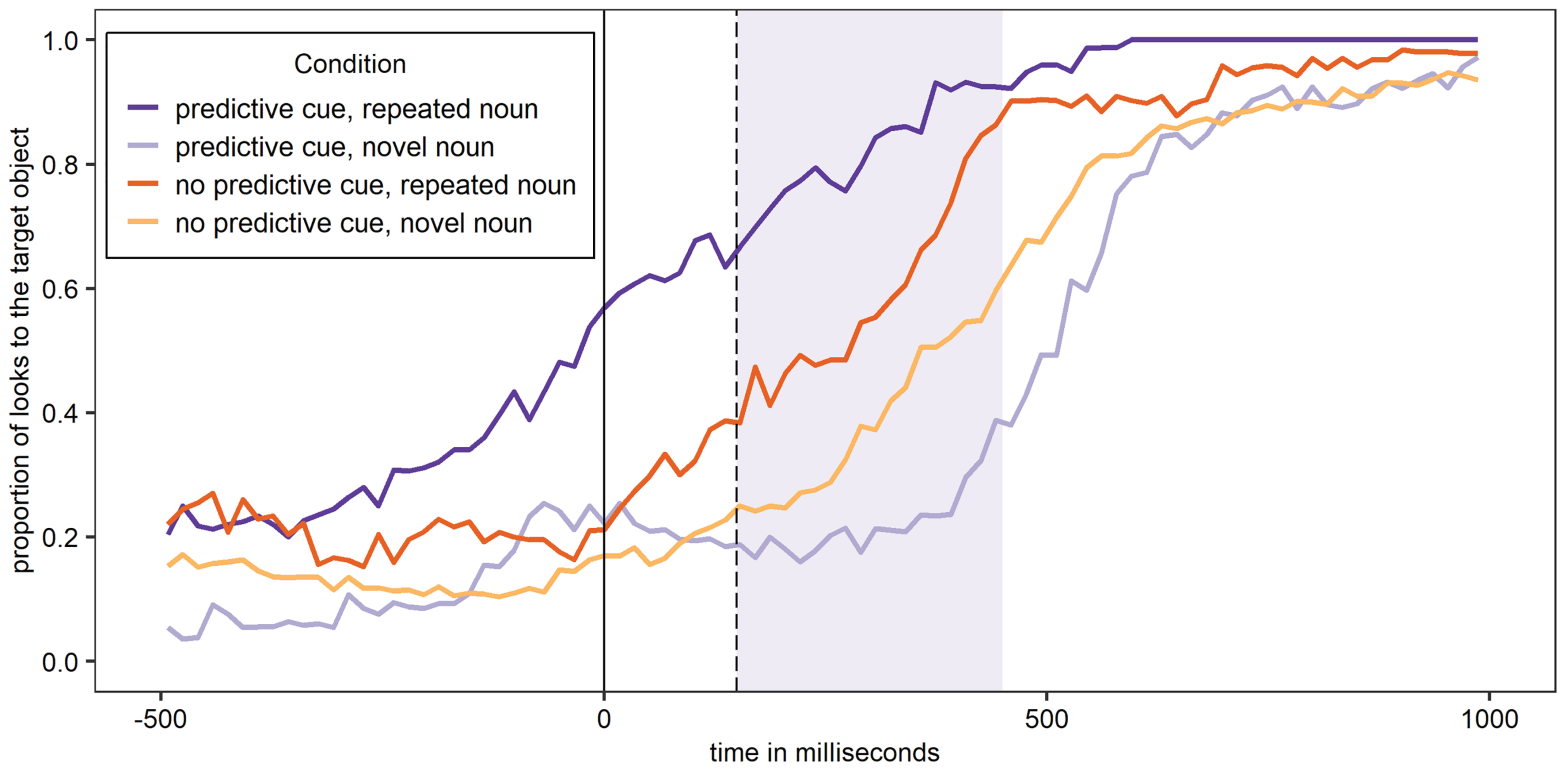
Proportion of looks over time to the target object across conditions in German (L1) after the training phase. The shaded area corresponds to the analysis window.

Figure 5 shows the proportion of looks to the target object over time for Experiment 2 and participants’ L1 German. The figure shows the same overall pattern as Figures 3 and 4, with most looks to the target object when a predictive L+H* cue was present and the noun was – as predicted – repeated, and the least looks to the target object when a predictive L+H* cue was present and the noun was – unexpectedly – not repeated.

The first analysis (Model 3A) tested if the experimental conditions and trial number significantly affected looks to the target object during the first 300 ms of processing the target noun in participants’ L1 German and following a training phase that increased the validity of the L+H* cue. The random effects structure of the final model included random intercepts for Participant and by-Participant random slopes for Predictive Cue, Repeated Noun, and Trial Number. The results are shown in Table 4. As for the German data of Experiment 1, there was a significant main effect of Repeated Noun, such that there were significantly more looks to the target noun overall if the noun was repeated. Importantly, the Predictive Cue by Repeated Noun interaction was again significant. To explore this interaction, separate models were fit for trials with repeated nouns and novel nouns, both with Predictive Cue as fixed factor, random intercepts for Participant, and by-Participant random slopes for Predictive Cue. There was a main effect of Predictive Cue for both repeated nouns (β = −1.00, *SE* = 0.23, z = −4.38, *p* < 0.001^***^) and novel nouns (β = 0.52, *SE* = 0.16, z = 3.37, *p* < 0.001^***^). This provides evidence for both facilitation of a repeated noun and inhibition of a novel noun following a predictive L+H* cue. Both Predictive Cue and Repeated Noun also significantly interacted with Trial Number, and the three-way interaction was significant. These interactions will not be further explored.

**Table 4:** Results of Models 3A, 3B, and 3C.

Fixed factors	Estimate	*SE*	*t-value*	*Value of p*
Model 3A				
Predictive Cue	0.09	0.13	0.70	=0.484
Repeated Noun	−0.89	0.19	−4.70	<0.001^***^
Trial Number	−0.09	0.07	−1.24	=0.217
Predictive Cue × Repeated Noun	0.57	0.04	16.25	<0.001^***^
Predictive Cue × Trial Number	0.08	0.03	2.25	=0.024^*^
Repeated Noun × Trial Number	0.26	0.03	7.75	<0.001^***^
Predictive Cue × Repeated Noun × Trial Number	0.07	0.03	2.13	=0.033^*^
Model 3B				
Inhibition Cost	−0.53	0.12	−4.45	<0.001^***^
Model 3C				
Predictive Cue	−24.07	5.77	−4.17	<0.001^***^
Repeated Noun	10.76	2.75	3.92	<0.001^***^
Trial Number	−0.56	0.13	−4.40	<0.001^***^
Inhibition Cost	−27.60	6.60	−4.18	<0.001^***^
Predictive Cue × Repeated Noun	−14.91	3.67	−4.06	<0.001^***^
Predictive Cue × Trial Number	0.78	0.17	4.59	<0.001^***^
Repeated Noun × Trial Number	−0.16	0.08	−1.87	=0.062
Predictive Cue × Repeated Noun × Trial Number	0.51	0.11	4.57	<0.001^***^

The second analysis (Model 3B) tested whether the Bayesian adaptation model significantly predicts looks to the target object during the first 300 ms of processing the target noun after participants had experienced a training session to increase the validity of the predictive cue. The random effects structure of the final model included random intercepts for Participant and by-Participant random slopes for Inhibition Cost. Table 4 shows that Inhibition Cost is indeed a significant predictor of participants’ looks to the target object. Again, the larger the inhibition cost, the fewer looks to the target object occurred, suggesting that participants did indeed inhibit the target noun if it was highly unexpected and updated their predictions accordingly.

Finally, the third analysis (Model 3C) tested whether the Bayesian adaptation model can predict looks to the target object beyond the experimental conditions and the trial number. The final model included no random effects. Table 4 shows that Inhibition Cost does indeed predict participants’ looks to the target object beyond the experimental conditions and the trial number. Again, the larger the inhibition cost, the fewer looks to the target object occurred. In order to see if Model 3C provided a better fit of the data compared to the same model without Inhibition Cost as a fixed effect, these two models were compared using the anova() function. Model 3C did indeed provide a significantly better fit of the data than the same model without Inhibition Cost (*p* < 0.001^***^; BIC Model 3C: 8549 vs. BIC model without Inhibition Cost: 8557; AIC Model 3C: 8488 vs. AIC model without Inhibition Cost: 8502).

#### L2 English

**Figure 6:**
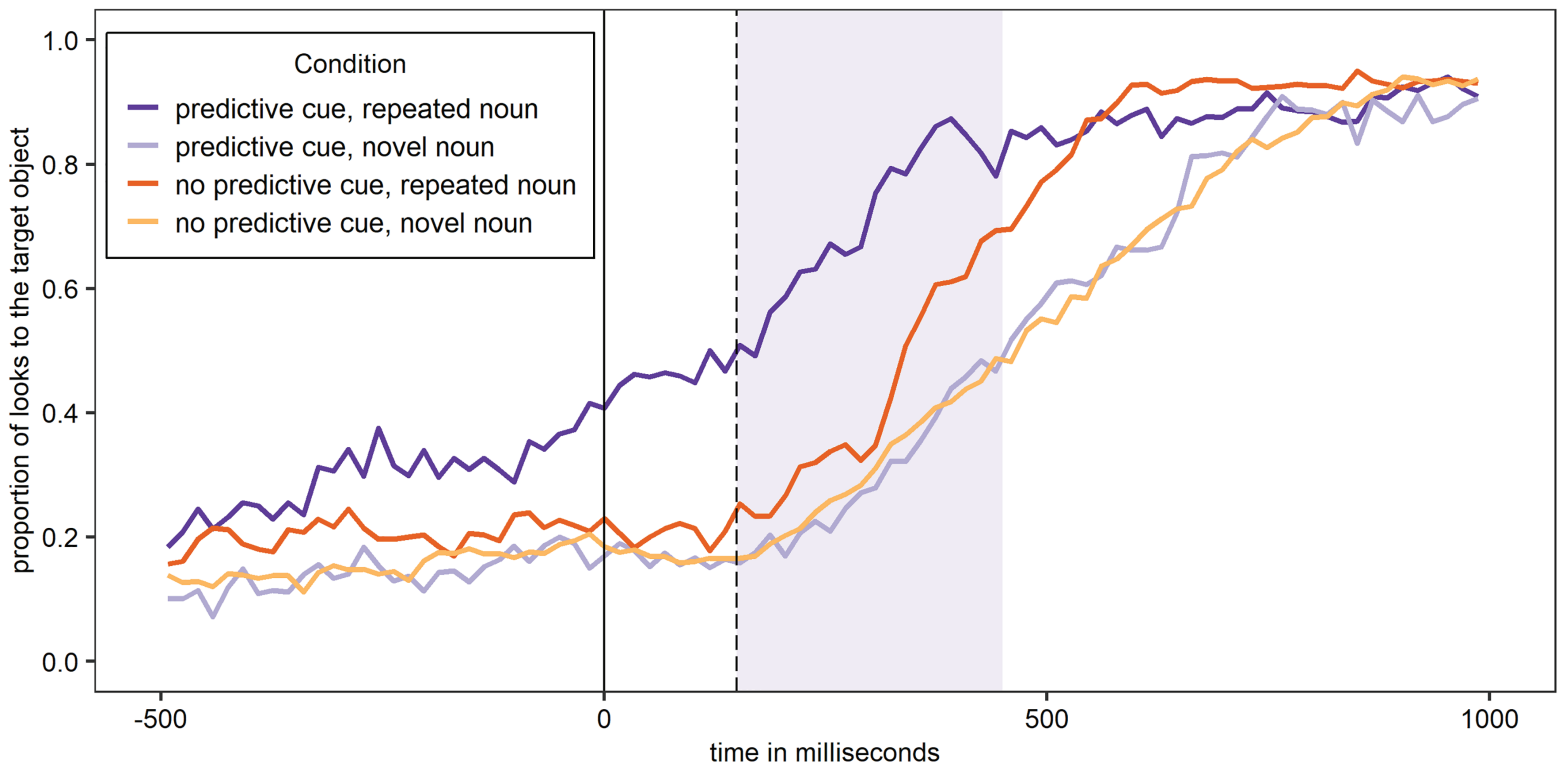
Proportion of looks over time to the target object across conditions in English (L2) after the training phase. The shaded area corresponds to the analysis window.

Figure 6 shows the proportion of looks to the target object over time for Experiment 2 and participants’ L2 English. The figure shows most looks to the target object when a predictive L+H* cue was present and the noun was – as predicted – repeated. In contrast, when the noun was not repeated, the proportion of looks to the target object was similar for trials with and without a predictive L+H* cue.

The first analysis (Model 4A) tested if the experimental conditions and trial number significantly affected looks to the target object during the first 300 ms of processing the target noun in participants’ L2 English and following a training phase that increased the validity of the L+H* cue. The random effects structure of the final model included random intercepts for Participant and by-Participant random slopes for Predictive Cue, Repeated Noun, Trial Number, and Repeated Noun by Trial Number. The results are shown in Table 5. As for Experiment 1, there was a significant main effect of Predictive Cue, but unlike the English data from Experiment 1, there were significantly more looks to the target object if there *was* a predictive L+H* cue compared to if there was not. As in all previous A models, there was also a significant main effect of Repeated Noun with significantly more looks to the target noun overall if the noun was repeated. Importantly, the Predictive Cue by Repeated Noun interaction was significant. To explore this interaction, separate models were fit for trials with repeated nouns and novel nouns, both with Predictive Cue as fixed factor, random intercepts for Participant, and by-Participant random slopes for Predictive Cue. There was a main effect of Predictive Cue for repeated nouns (β = −0.73, *SE* = 0.13, z = −5.70, *p* < 0.001^***^), providing evidence for facilitation of a repeated noun following a predictive L+H* cue. In contrast, Predictive Cue was not a significant factor for novel nouns (β = 0.06, *SE* = 0.19, z = 0.34, *p* = 0.736). There is thus no evidence for inhibition of a novel noun following a predictive L+H* cue. Predictive Cue also significantly interacted with Trial Number, but this interaction will not be further explored here.

**Table 5:** Results of Models 4A and 4B.

Fixed factors	Estimate	*SE*	*t-value*	*Value of p*
Model 4A				
Predictive Cue	−0.28	0.13	−2.14	=0.032^*^
Repeated Noun	−0.57	0.12	−4.72	<0.001^***^
Trial Number	−0.11	0.12	−0.84	=0.403
Predictive Cue × Repeated Noun	0.42	0.03	12.34	<0.001^***^
Predictive Cue × Trial Number	−0.07	0.03	−2.09	=0.037^*^
Repeated Noun × Trial Number	0.15	0.17	0.88	=0.378
Predictive Cue × Repeated Noun × Trial Number	0.06	0.03	1.61	=0.108
Model 4B				
Inhibition Cost	−0.20	0.13	−1.52	=0.129

The second analysis (Model 4B) tested whether the Bayesian adaptation model significantly predicts looks to the target object during the first 300 ms of participants processing the target noun in their L2 English and after participants had experienced a training session to increase the validity of the predictive cue. The random effects structure of the final model included random intercepts for Participant and by-Participant random slopes for Inhibition Cost. Table 5 shows that Inhibition Cost is not a significant predictor of participants’ looks to the target object in their L2 English.

### Summary of the Main Results

Table 6 summarizes the main results of the analyses. The column for the A models shows the significant results of the experimental conditions (excluding significant results involving trial number). The column shows that there was a main effect of Repeated Noun across all experiments with significantly more looks to the target noun when the noun was repeated compared to when it was not. There was also a main effect of Predictive Cue in both L2 English experiments, but not in the L1 German experiments. However, the main effect of Predictive Cue went in opposite directions across the two L2 English experiments. Specifically, there were more looks to the target noun for adjectives *not* carrying a L+H* accent in Experiment 1 (without training), but more looks to the target noun for adjectives carrying a L+H* accent in Experiment 2 (with training). Finally, there was a significant Predictive Cue by Repeated Noun interaction across all experiments. Importantly, the two L1 German experiments showed consistent significant inhibition for a novel noun following a L+H* accent compared to following no L+H* accent. In contrast, in the case of the L2 English, only Experiment 1, but not Experiment 2, showed significant inhibition for a novel noun following a L+H* accent compared to following no L+H* accent. The column for the B and C models additionally shows that, for the two L1 German experiments, the Bayesian adaptation model was a significant predictor of looks to the target noun both by itself and beyond the experimental conditions. In contrast, the Bayesian adaptation model only significantly predicted looks to the target noun by itself (and beyond the experimental conditions) for the L2 English Experiment 1, but not for the L2 English Experiment 2.

**Table 6:** Summary of the main analysis results.

Experiment	A models: significant results of experimental conditions (excluding results involving trial number)	B models: significant predictor?	C models: significant predictor?
L1 German, no training	Main effect of Repeated Noun: more looks to target noun if noun was repeated vs. not repeated Predictive Cue by Repeated Noun interaction: no facilitation for repeated noun after L+H*, but inhibition for novel noun after L+H*	Yes	Yes
L2 English, no training	Main effect of Predictive Cue: more looks to target noun if no L+H* vs. L+H* Main effect of Repeated Noun: more looks to target noun if noun was repeated vs. not repeated Predictive Cue by Repeated Noun interaction: facilitation for repeated noun after L+H* and inhibition for novel noun after L+H*	Yes	Yes
L1 German, training	Main effect of Repeated Noun: more looks to target noun if noun was repeated vs. not repeated Predictive Cue by Repeated Noun interaction: facilitation for repeated noun after L+H* and inhibition for novel noun after L+H*	Yes	Yes
L2 English, training	Main effect of Predictive Cue: more looks to target noun if L+H* vs. no L+H* Main effect of Repeated Noun: more looks to target noun if noun was repeated vs. not repeated Predictive Cue by Repeated Noun interaction: facilitation for repeated noun after L+H*, but no inhibition for novel noun after L+H*	No	N/A

## Discussion

The current study analyzed eye movement data during the processing of a target noun that was or was not preceded by a L+H* accent that would lead participants to predict a repeated noun. The following sections will discuss the results of both experiments in relation to predictive processing and adaptation in the face of prediction error.

### Experiment 1: No Training

The L1 data from Experiment 1 showed evidence for significant inhibition of an unpredicted target noun, as evidenced by fewer looks to the target noun following a L+H* cue compared to no L+H* cue. Together with the results of Foltz (2020) that participants did predict a repeated noun before bottom-up information from the noun had come in, these results suggest that a L+H* accent lead participants to predictively pre-update a repeated-noun referent, which caused significant inhibition when the bottom-up input did not confirm this pre-updated referent. However, there was no evidence for facilitation of a predicted target noun during the processing of the noun. While participants looked more at a repeated target noun following a L+H* accent compared to following no L+H* accent, this difference failed to reach significance. One possibility for the lack of a significant effect is the size of the analysis window, which was chosen to be relatively large at 300 ms to make sure that it reflected unambiguous processing of the target noun. However, since participants were already near ceiling toward the end of the analysis window, the analysis may not have detected a facilitation effect from the presence of a L+H* predictive cue.

The L2 data from Experiment 1 showed evidence for both significant facilitation of a predicted target noun and significant inhibition of an unpredicted target noun. Together with the results of Foltz (2020) that participants did not predict a repeated noun before receiving bottom-up information from the noun, the current results suggest that participants may indeed engage in predictive processing, but are overall slower in their processing, such that measurable effects do not surface until bottom-up information is already available. This would be consistent with resource-deficit accounts of L2 processing (McDonald, 2006; Hopp, 2009), which assume that L2 processing is fundamentally similar to L1 processing and that observed differences in L1 and L2 processing are due to resource limitations. Specifically, it seems that participants use prosodic cues more slowly in the L2, possibly because – as intermediate to advanced English speakers – their processing routines are less automatic (Hopp, 2009) or their lexical access is slower (Miller, 2014). In line with this, we may expect bilinguals who are highly proficient to be overall faster in their processing and show both L+H*-driven prediction of a repeated noun before bottom-up information from the noun arrives as well as significant inhibition of an unpredicted target noun once bottom-up information from the noun is available.

For both L1 and L2 processing of Experiment 1, the Bayesian adaptation model was a significant predictor of the eye movement data both by itself and beyond the experimental conditions and the number of trials encountered. This suggests that participants adapt to strong prediction errors by updating their beliefs about the predictive validity of the L+H* cue and adapting the strength of their subsequent predictions accordingly. The results provide further evidence for the important role of prediction error in language processing and adaptation (Chang et al., 2006; Dell and Chang, 2014). The results also present initial evidence that a trial-by-trial Bayesian adaptation model can model both L1 and L2 eye-tracking data during language processing and provide further evidence that L2 processing is fundamentally similar to L1 processing (Hopp, 2009). It should be noted though that the current study explored L2 prosodic processing for two closely related languages that use the same prosodic cue (a L+H* accent) to mark a contrast. Since L2 processing tends to be more nativelike when the L1 and L2 are similar (Sabourin and Stowe, 2008; Foucart and Frenck-Mestre, 2011; Dussias et al., 2013), L2 processing when the L1 and L2 are less similar might be quite different from the results found here. Specifically, it is possible that participants in the current study had sufficient resources to adapt to strong prediction errors in their L2 over the course of the experiment because the similarity of their L1 and L2 allowed them to transfer processing routines from the L1 to the L2 and free up processing resources for prediction error tracking.

### Experiment 2: With Training

The L1 data from Experiment 2 show that, following a training session that increased the predictive validity of the L+H* cue, participants experienced both significant facilitation of a predicted target noun (unlike the L1 data for Experiment 1) and significant inhibition of an unpredicted target noun. Thus, with increased predictive validity of the L+H* cue at the beginning of the experiment, both facilitation of a predicted target and inhibition of an unpredicted target occur. In line with the L1 data in Experiment 1, the Bayesian adaptation model was a significant predictor of the eye movement data both by itself and beyond the experimental conditions and the number of trials encountered. Together with the results of Foltz (2020) that participants did predict a repeated noun before bottom-up information from the noun had come in, these results again suggest that a L+H* accent lead participants to predictively pre-update a repeated-noun referent, which caused significant inhibition when the bottom-up input did not confirm this pre-updated referent.

The L2 data from Experiment 2 show that, following training, participants experienced only significant facilitation of a predicted target noun, but no inhibition of an unpredicted target noun. This contrasts with the L2 data from Experiment 1. In addition, the Bayesian adaptation model did not significantly predict the eye movement patterns. One possible explanation for the differences in L2 processing across the two experiments is that participants have more difficulties tracking changes in the statistics of the input. Specifically, native listeners adapt their processing to the specific prosodic properties of individual speakers’ speech (Roettger and Rimland, 2020). In the current study, the speaker was internally inconsistent. Specifically, participants first experienced the speaker using the L+H* cue inconsistently in Experiment 1, such that a L+H* accent preceded a repeated noun as often as a novel noun. After a short break, participants then experienced the training session of Experiment 2, where the same speaker used the L+H* cue highly consistently, such that a L+H* accent always preceded a repeated noun. The training session was immediately followed by the second experiment, where the same speaker again used the L+H* cue inconsistently. Detecting and adapting to these changes in how the same speaker uses L+H* accents differently over time requires quite detailed tracking of the reliability of the L+H* cue, and intermediate to advanced L2 listeners may be limited in the resources that they can allocate to such detailed tracking. In this case, we might expect that highly proficient L2 listeners may better be able to track such changes in cue reliability and might show processing that is more in line with the L1 results found here. In contrast, L2 listeners with a L1 that is less similar to their L2 might have additional difficulties tracking cue reliability over time. Alternatively, L2 listeners may be slower to “reverse course,” such that once they consider a cue to be unreliable, it takes more positive evidence than was provided in the training session for them to begin to rely on the L+H* cue again. Such difficulties in tracking changes in the statistics of the input may explain why there is no evidence for inhibition when encountering an unpredicted noun in the L2 in Experiment 2 and why the Bayesian adaptation model does not account for the L2 eye movement data of Experiment 2. Thus, L2 processing may differ from L1 processing when the statistical properties of cues change in a seemingly random manner (from consistent to inconsistent and back to consistent), which may require more resources to track.

The L2 data from Experiment 2 also need to be reconciled with the results of Foltz (2020), which showed that participants predicted a repeated noun following a L+H* accent before bottom-up information from the noun had come in. Thus, participants did indeed engage in predictive processing, which should have led to inhibition for a novel noun after a L+H* accent. One possible explanation is that participants only pre-activated and did not pre-update the predicted noun, thus resulting in prediction, but not inhibition. Ness and Meltzer-Asscher (2018) mention that evidence of predictive processing in an eye-tracking task, as found during the processing of the adjective in Foltz (2020), can only provide evidence for pre-activation, not for pre-updating. So, it is possible that participants used the high-level event hypotheses to activate linguistic representations before bottom-up input reached these levels of representation, leading to prediction. But participants may not have included this predicted content in the linguistic representation that they were building in working memory before receiving bottom-up input. This would explain the combination of evidence for prediction during the processing of the adjective, as found in Foltz (2020), without evidence for inhibition during the processing of the noun, as found here. Pre-activation leads to pre-updating for strong predictions, so it is possible that the cue changes in a seemingly random manner led participants to not commit to their predictions.

Another possibility is that participants were less driven by the predictive L+H* cue in their L2 and more by aspects of the visual display or that participants began relying more on aspects of the visual display as prosodic cues began to change in a seemingly random manner. Specifically, both the current data and the results of Foltz (2020) show either numerically or significantly more looks to the repeated noun, regardless of pitch accent cue. In addition, as would be expected for natural productions, color adjectives with a L+H* accent were significantly longer than color adjectives without a L+H* accent (484 vs. 331 ms for German; 338 vs. 268 ms for English). Participants thus had information about the color of the second object earlier for trials with a L+H* accent compared to without. This would allow participant to narrow down the target object from five (all except the target of the first instruction) to two (the two objects in the mentioned color) earlier for trials with a L+H* accent compared to without. If participants generally preferred a repeated noun and narrowed in on color information of the target object earlier in L+H* trials, then this may explain why there seems to be evidence for predictive processing during the processing of the adjective, without evidence of inhibition during the processing of the noun.

## Conclusion

The current study used a Bayesian adaptation model to model trial-by-trial adaptation to L+H* cues in bilingual participants’ L1 and L2 processing. The study employed such a model to eye-tracking data and showed that it does indeed account for participants’ L1 processing across two experiments that differed in the validity of the predictive cue. In contrast, the results suggest that the model accounted for the L2 processing data of only Experiment 1, but not Experiment 2. Overall, the current study provides further evidence for the important role of prediction error in processing and adaptation, such that interlocutors with a similar language background would in real-life communication be expected to adapt their predictive processing to the prosodic cues available in the input and to the reliability of these cues in a similar way to how listeners adapted in the current study. The results also suggest that Bayesian adaptation models are a useful avenue to model L2 processing, but that they seem to model L2 processing less robustly in the face of frequently changing statistical probabilities.

## Data Availability Statement

The datasets presented in this study can be found at: https://osf.io/xey27/.

## Ethics Statement

The studies involving human participants were reviewed and approved by the Ethics committee of the University Hospital Münster. The patients/participants provided their written informed consent to participate in this study.

## Author Contributions

AF oversaw the data collection, conducted the analyses, and wrote the paper.

### Conflict of Interest

The author declares that the research was conducted in the absence of any commercial or financial relationships that could be construed as a potential conflict of interest.
